# Targeted NGS Platforms for Genetic Screening and Gene Discovery in Primary Immunodeficiencies

**DOI:** 10.3389/fimmu.2019.00316

**Published:** 2019-04-11

**Authors:** Cristina Cifaldi, Immacolata Brigida, Federica Barzaghi, Matteo Zoccolillo, Valentina Ferradini, Davide Petricone, Maria Pia Cicalese, Dejan Lazarevic, Davide Cittaro, Maryam Omrani, Enrico Attardi, Francesca Conti, Alessia Scarselli, Maria Chiriaco, Silvia Di Cesare, Francesco Licciardi, Montin Davide, Francesca Ferrua, Clementina Canessa, Claudio Pignata, Silvia Giliani, Simona Ferrari, Georgia Fousteri, Graziano Barera, Pietro Merli, Paolo Palma, Simone Cesaro, Marco Gattorno, Antonio Trizzino, Viviana Moschese, Loredana Chini, Anna Villa, Chiara Azzari, Andrea Finocchi, Franco Locatelli, Paolo Rossi, Federica Sangiuolo, Alessandro Aiuti, Caterina Cancrini, Gigliola Di Matteo

**Affiliations:** ^1^Unit of Immune and Infectious Diseases, University Department of Pediatrics (DPUO), Scientific Institute for Research and Healthcare (IRCCS) Childrens' Hospital Bambino Gesù, Rome, Italy; ^2^San Raffaele Telethon Institute for Gene Therapy (SR-Tiget), IRCCS San Raffaele Scientific Institute, Milan, Italy; ^3^Pediatric Immunohematology and Bone Marrow Transplantation Unit, Scientific Institute for Research and Healthcare (IRCCS) San Raffaele Scientific Institute, Milan, Italy; ^4^Department of Systems Medicine, University of Rome Tor Vergata, Rome, Italy; ^5^Department of Biomedicine and Prevention, University of Rome Tor Vergata, Rome, Italy; ^6^Vita Salute San Raffaele University, Milan, Italy; ^7^Center for Translational Genomics and BioInformatics, San Raffaele Scientific Institute, Milan, Italy; ^8^Division of Immunology and Rheumatology, Department of Paediatric Infectious Diseases, Regina Margherita Children's Hospital, University of Turin, Turin, Italy; ^9^Pediatric Immunology, Department of Health Sciences, University of Florence, Florence, Italy; ^10^Meyer Children's Hospital, Florence, Italy; ^11^Department of Translational Medical Sciences, University of Naples Federico II, Naples, Italy; ^12^Department of Molecular and Translational Medicine, A. Nocivelli Institute for Molecular Medicine, University of Brescia, Brescia, Italy; ^13^Unit of Medical Genetics, St. Orsola-Malpighi University Hospital, University of Bologna, Bologna, Italy; ^14^Division of Immunology Transplantation and Infectious Diseases (DITID), Diabetes Research Institute (DRI) IRCCS San Raffaele Scientific Institute, Milan, Italy; ^15^Pediatric Department, San Raffaele Scientific Institute, Milan, Italy; ^16^Department of Onco-Hematology and Cell and Gene Therapy, Scientific Institute for Research and Healthcare (IRCCS) Childrens' Hospital Bambino Gesù, Rome, Italy; ^17^Paediatric Hematology-Oncology, “Ospedale della Donna e del Bambino”, Verona, Italy; ^18^Center for Autoinflammatory Diseases and Immunodeficiencies, IRCCS Giannina Gaslini, Genoa, Italy; ^19^Department of Pediatric Hematology and Oncology, “ARNAS Civico Di Cristina Benfratelli” Hospital, Palermo, Italy; ^20^Pediatric Immunopathology and Allergology Unit, University of Rome Tor Vergata Policlinico Tor Vergata, Rome, Italy; ^21^Milan Unit, National Research Council (CNR) Institute for Genetic and Biomedical Research (IRGB), Milan, Italy; ^22^Humanitas Clinical and Research Institute, Rozzano, Italy; ^23^Department of Pediatric Hematology and Oncology, Scientific Institute for Research and Healthcare (IRCCS) Childrens' Hospital Bambino Gesù, University of Rome La Sapienza, Rome, Italy

**Keywords:** primary immunodeficiencies, Next Generation Sequencing, gene panels, Ion Torrent, Haloplex

## Abstract

**Background:** Primary Immunodeficiencies (PIDs) are a heterogeneous group of genetic immune disorders. While some PIDs can manifest with more than one phenotype, signs, and symptoms of various PIDs overlap considerably. Recently, novel defects in immune-related genes and additional variants in previously reported genes responsible for PIDs have been successfully identified by Next Generation Sequencing (NGS), allowing the recognition of a broad spectrum of disorders.

**Objective:** To evaluate the strength and weakness of targeted NGS sequencing using custom-made Ion Torrent and Haloplex (Agilent) panels for diagnostics and research purposes.

**Methods:** Five different panels including known and candidate genes were used to screen 105 patients with distinct PID features divided in three main PID categories: *T cell defects, Humoral defects* and *Other PIDs*. The Ion Torrent sequencing platform was used in 73 patients. Among these, 18 selected patients without a molecular diagnosis and 32 additional patients were analyzed by Haloplex enrichment technology.

**Results:** The complementary use of the two custom-made targeted sequencing approaches allowed the identification of causative variants in 28.6% (*n* = 30) of patients. Twenty-two out of 73 (34.6%) patients were diagnosed by Ion Torrent. In this group 20 were included in the SCID/CID category. Eight out of 50 (16%) patients were diagnosed by Haloplex workflow. Ion Torrent method was highly successful for those cases with well-defined phenotypes for immunological and clinical presentation. The Haloplex approach was able to diagnose 4 SCID/CID patients and 4 additional patients with complex and extended phenotypes, embracing all three PID categories in which this approach was more efficient. Both technologies showed good gene coverage.

**Conclusions:** NGS technology represents a powerful approach in the complex field of rare disorders but its different application should be weighted. A relatively small NGS target panel can be successfully applied for a robust diagnostic suspicion, while when the spectrum of clinical phenotypes overlaps more than one PID an in-depth NGS analysis is required, including also whole exome/genome sequencing to identify the causative gene.

## Introduction

Primary immunodeficiencies (PIDs) are a phenotypically and genetically heterogeneous group of more than 300 monogenic inherited disorders resulting in immune defects that predispose patients to infections, autoimmune disorders, lymphoproliferative disease, and malignancies (1–3). PIDs with a more severe phenotype lead to life-threatening infections and life-limiting complications that require a prompt and accurate diagnosis in order to initiate lifesaving therapy (4, 5). Phenotypic and genotypic heterogeneity of PIDs make genetic diagnosis often complex and delayed. Indeed, more than one genotype might cause similar clinical phenotypes, but identical genotypes will not often produce the same phenotype and finally clinical penetrance may be different (6–9). The characterization of PID-associated genes is expected to significantly contribute to define the molecular events governing immune system development and will provide new insights into the pathogenesis of PIDs. Molecular genetic testing is also a useful tool for the diagnosis of PIDs in atypical cases (6, 10).

Despite the progress in the genetic characterization of PIDs, many patients still lack a molecular diagnosis. A better understanding of the genetic and immune defects of patients is critical to develop therapeutic strategies aimed at changing the clinical course of the disease and to guarantee an appropriate genetic counseling allowing the identification of PID patients before the onset of the disease (11–13). The application of Next Generation Sequencing (NGS) to PIDs has been a revolution and it has accelerated the discovery and identification of novel disease-causing genes and the genetic diagnosis of patients with monogenic inborn errors of immunity (7, 8, 14–16). Targeted gene-panel sequencing (17–21), whole exome sequencing (WES) (22, 23) or whole genome sequencing (WGS) (24) approaches can rapidly identify candidate gene variants in an increasing number of genetically undefined diseases (17, 24) and are widely used in several laboratories for the diagnosis of PIDs (10). WGS also offers the opportunity to find causative variants in the structural regions of a given gene. These tools increase the amount of data analysis that can identify causative genes in both clinically defined and atypical diseases. Nonetheless, delay in diagnosis can be caused by the huge amount of data retrieved from whole sequencing, increased costs sustained by clinical laboratories and the requirement of trained personnel to validate variants (7, 8, 22). An increased depth of the sequencing coverage is generally obtained using targeted gene panels, in favor of a high accuracy, amelioration of sensitivity and management of datasets, reducing the time of analysis, the costs and the interpretation of results, thus accelerating the diagnosis for the majority of PIDs (14, 16–18). On the other hand, the usefulness of targeted exome sequencing approach for the identification of PID patients has been demonstrated, with accurate detection of point mutations and exonic deletions in patients with either known or unknown genetic diagnosis (7, 8).

In this study, we report the clinical and molecular characterization of 105 PID patients presenting with either typical SCID/CID or with overlapping PID phenotypes. Differently from other studies (20, 21, 25), most patients enrolled in this work had non-consanguineous parents. Two targeted sequencing approaches were compared to test the ion torrent reliability in diagnostics and Haloplex Target Enrichment System in diagnostics and for research purposes. **Three** diagnostic panels including known disease genes had been developed for the Ion Torrent platform (ThermoFisher). The Haloplex panels comprised well-defined PID genes (>300) and candidate genes associated with PIDs due to their expression and function in critical immune-pathways (1, 3). This work underlines how targeted NGS panels allow a high-throughput low-cost pipeline to identify the molecular bases of PIDs and are sensitive and accurate diagnostic tools for simultaneous mutation screening of known or putative PID-related genes.

## Materials and Methods

### Patients

We report the clinical and molecular characterization of 105 PID patients mainly referred to three centers (2 in Rome and 1 in Milan) participating in the Italian network of PIDs (IPINET) and part of The European Reference Network on immunodeficiency, autoinflammatory, and autoimmune diseases (ERN RITA). Nine of these patients have been enrolled in the pCID study (DRKS00000497). Data were obtained from year 2014 to 2017.

Ion Torrent and/or Haloplex panels were applied for the analysis of samples and compared. Six patients previously diagnosed by Sanger sequencing were included in the study (**Table 2A**) as internal positive controls. The Ion Torrent panels were used for the analysis of 73 patients with suspicion of PID. Among this group, 18 patients, still remaining without a molecular diagnosis and 32 additional patients, were tested by Haloplex panels (Target Enrichment System for Illumina platform). The work was conducted in accordance with the ethical standards of the institutional research committee and with the 1964 Helsinki declaration and its later amendments or comparable ethical standards. Informed consent, approved by the Ethical Committee of the Children's Hospital Bambino Gesù, San Raffaele Hospital (TIGET06, TIGET09) and Policlinico Tor Vergata, was obtained from either patients or their parents/legal guardians, if minors. Patients and their clinical and immunological features are reported in Table 1.

**Table 1:** Clinical, immunological and molecular features of PID patients.

**PID ID**	**AGE AT PRESENTATION**	**GENDER**	**ADMITTING CLINICAL DIAGNOSIS**	**NGS PLATFORM**	**GENETIC DIAGNOSIS**	**NEUTRAL VARIANTS AND VUS**	**OPPORTUNISTIC/RECURRENT INFECTIONS**	**IMMUNEDYSREGULATION/ MALIGNANCIES/ OTHERS**	**IMMUNOPHENOTYPE**
PID 1	BIRTH	M	OMENN SYNDROME	ION TORRENT PANEL 1	***RAG1***	**	CHRONIC CMV VIREMIA, PNEUMOCYSTOSIS, HERPETIC KERATITIS	TUBULE INTERSTITIAL NEPHRITIS WITH LYMPHO-MONOCYTE INFILTRATE	T+, B-, NK+
PID 2	2 mo	M	SCID	ION TORRENT PANEL 1	***RAG2***	**	EBV AND ADENOVIRUS POST HSCT		T+ (↓CD4 , ↓CD8), B-, ↑NK, ↓IgM, ↓IgA
PID 3	5 mo	F	SCID	ION TORRENT PANEL 1	***RAG2***	**	ADENOVIRUS		T-, B-, NK+
PID 4	2mo	M	SCID	ION TORRENT PANEL 1	***RAG1***	**	CHRONIC CMV VIREMIA		T+, B-, NK+, ↓IgM
PID 5	5mo	M	SCID	ION TORRENT PANEL 1	***IL2RG***	**	ADENOVIRUS, HEPATITIS, ENTEROBACTHER CLOACAE; CANDIDA	DERMATITIS (BOLLOUS TYPE)	T-, B-,↑NK
PID 6	4mo	M	SCID	ION TORRENT PANEL 1	***JAK3***	**	INTERSTITIAL PNEUMONIA, PNEUMOCYSTOSIS		T-, B+, NK-, HYPGAMMAGLOBULINEMIA
PID 7	4mo	M	SCID	ION TORRENT PANEL 1-2			INTERSTITIAL LUNG DISEASE; URI; LRI	HEPATOSPLENOMEGALY, AIHA, ITP	T+ (ABSENT NAIVE and RTE, ↑γδ), B+, NK+
PID 8	1 y	M	SCID	ION TORRENT PANEL 1-2				HEPATOSPLENOMEGALY, AIHA, ITP, VERTEBRAL WEDGING AND OSTEOPENIA	T (ABSENT NAIVE and RTE, ↑γδ), B+(↑UNSWITCHED MEMORY), NK+
PID 9	9mo	F	SCID	ION TORRENT PANEL 1-2			GLUTEAL ABSCESS	CHRONIC DIARRHEA	T+ (↑CM CD4 , ↑γδ), B-, NK-
PID 10	2mo	F	SCID	ION TORRENT PANEL 1-2			POST-NATAL CMV INFECTION; URI;LRI; NEONATAL SEPSIS		T+, B (ABSENT SWITCHED MEMORY), NK+
PID 11	na	F	SCID	ION TORRENT PANEL 1-2			CHRONIC VZV VIREMIA	THROMBOCYTOPENIA	T- (↓ CD4), B+, NK+
PID 12	1y	M	SCID	ION TORRENT PANEL 1-2/ HALOPLEX PANEL 2	***ADA***	**	URI		T-, B-, NK+
PID 13	2y	M	SCID	ION TORRENT PANEL 1-2/ HALOPLEX PANEL 2		*CECR1*	PENUMONIA; CANDIDIASIS	GASTROENTERITIS, HyperIgE; LYELL SYNDROME, CARDIAC ARREST OF UNKNOWN ORIGIN; ILEOILEAL INTUSSUSCEPTION	LOW T, B-, ↑NK+, ↑IgM, ↓IgA, ↑IgE
PID 14	5mo	M	SCID	ION TORRENT PANEL 1-2			CHRONIC CMV AND EBV VIREMIA		↓T (ABSENT NAIVE CD4 and CD8, ↑γδ), B+, ↑NK
PID 15	1y	F	SCID	ION TORRENT PANEL 1-2	***ADA***	**	LRI		T-, B-, NK+, ↓IgM
PID 16	8 mo	M	leaky SCID	ION TORRENT PANEL 1-2	***IL2RG***		CHRONIC CMV VIREMIA, SEVERE CMV INTERSTITIAL PNEUMONIA	PNEUMONIA, DERMATITIS, GROWTH FAILURE	T+/-, B-, NK+
PID 17	1y	M	SCID	ION TORRENT PANEL 1	***IL2RG***	**	CHRONIC CMV VIREMIA, BRONCHIOLITIS, UTI, ROTAVIRUS ENTERITIS	HEPATOSPLENOMEGALY, HLH	↓T (ABSENT NAIVE CD4 and CD8), B-, NK+
PID 18	4d (DIED)	M	SCID	ION TORRENT PANEL 1	***IL2RG***	**	CMV, PNEUMOCYSTIS JIROVECI PNEUMONIA		T-, B+, ↓NK
PID 19	5mo	M	SCID	ION TORRENT PANEL 1	***IL2RG***	**	STAPHYLOCOCCUS HAEMOLYTICUS; ASPERGILLUS, BCGITIS		T-, ↑B+, ↓NK+, ↓IgM, ↓IgA
PID 20	1.8y	F	SCID	ION TORRENT PANEL 1-2	***RAG1***	**	LTI	HEPATOSPLENOMEGALY	T-, B-, NK+, ↑IgG, ↓IgM, ↓IgA, ↓IgE
PID 21	1y	M	SCID	ION TORRENT PANEL 1-2	***CD3D***	**	RHINOVIRUS, MYCOBACTERIUM		T (↓NAIVE CD4, ABSENT CD8, ↑γδ), B+, NK+
PID 22	1.3y	M	SCID	ION TORRENT PANEL 1	***RAG1***	**	LRI, ADENOVIRUS, ROTAVIRUS ENTERITIS, PSEUDOMONAS AERUGINOSA		T-, B-, NK+, ↓IgM, ↓IgA, ↓IgE
PID 23	11mo	F	SCID	ION TORRENT PANEL 1	***JAK3***	**	CHRONIC HHV-6 VIREMIA, CANDIDA ALBICANS, ROTAVIRUS, CORONAVIRUS 229E,		T-, B+, ↑NK, ↓IgG, ↓IgM, ↓IgA
PID 24	4mo	F	SCID	ION TORRENT PANEL 1	***JAK3***	**	LRI, CANDIDA ALBICANS, RHINOVIRUS		T-, B+, ↑NK, ↓IgE
PID 25	na	M	SCID	HALOPLEX PANEL 1	***IL7R***	**	na	na	T-, B+, NK+
PID 26	1y	M	CID	HALOPLEX PANEL 1				AIHA	T-, B-, NK+, ↓IgM, ↓IgA
PID 27	5	F	CID	ION TORRENT PANEL 1-2/ HALOPLEX PANEL 1			IR, EPIDERMODYSPLASIA VERRUCIFORMIS (HPV-8 WARTS); URI	MILD MYELODYSPLASIA, SEVERE HEPATIC STEATOSIS	T (↓NAIVE and ↓RTE), B+, NK+
PID 28	3	F	CID	ION TORRENT PANEL 1-2/ HALOPLEX PANEL 1			CHRONIC EBV VIREMIA, URI, PNEUMONIA	ENTEROPATHY; CHRONIC-PANCREATITIS; ANA/ANCA+	T (↓NAIVE), B+, NK+
PID 29	1 y	M	CID	ION TORRENT PANEL 1	***RAG1***	**	CHRONIC CMV AND EBV VIREMIA, HAEMOPHILUS INFLUENZAE AND BOCAVIRUS RESPIRATORY INFECTION, LONG-LASTING ROTAVIRUS DIARRHEA	THROMBOCYTOPENIA, AHIA , SEVERE HEPATOSPLENOMEGALY, WITH LIVER FAILURE	↓T, B+, NK+, ↑IgE, ↑IgM, ↑IgG
PID 30	3	M	CID	ION TORRENT PANEL 1-2			URI	DERMATITIS; ANA+	↓T (↓NAIVE CD4), B+, NK+
PID 31	3	F	CID	ION TORRENT PANEL 1	***RAG1***	**	CHRONIC HHV-6, CMV,EBV VIREMIA; URI, LRI	AHIA	T+, ↓ B, NK+, ↓IgM, ↓IgA
PID 32	3y	F	CID	ION TORRENT PANEL 1-2/ HALOPLEX PANEL 1	**	**	CHRONIC EBV VIREMIA, URI	HODGKIN LYMPHOMA; ENTEROPATHY	T (↓NAIVE), B-, NK+
PID 33	10y	M	CID	ION TORRENT PANEL 2	**	**	CHRONIC EBV VIREMIA, URI-LRI	LYMPHADENOPATHY, URTICARIA, LONG-COURSE DIARROHEA /LYMPHATIC HYPERPLASIA	T+, B-, NK+
PID 34	1y	F	CID	ION TORRENT PANEL 1	**	**		SEVERE DERMATITIS, DIARRHEA, INTERSTITIOPATHY	T (↓CD8), ↑B+, NK+
PID 35	11y	F	CID	HALOPLEX PANEL 1				COLITIS, GH DEFICIENCY	LYMPHOPENIA, ↓T, ↓B, ↓NK (UNDER AZA)
PID 36	15 mo	F	CID	ION TORRENT PANEL 1-2	***IL7R***	**	CHRONIC EBV VIREMIA, URI (recurrent)	THROMBOCYTOPENIA,SEVERE DERMATITIS, GROWTH RETARDATION,	HYPERGAMMAGLOBULINEMIA (maternal engraftment)
PID 37	1mo	M	CID	HALOPLEX PANEL 2	***ARPC1B***	**	WARTS, RECURRENT INFECTIONS	VASCULITIS, LYMPHADENOPATHY, ECZEMA, HYPOGAMMAGLOBULINEMIA, HYPER IgE, THROMBOCYTOPENIA, LUNG DISEASE, BRONCHIECTASIS	↓T, B+, ↓NK, ↓IgG , ↓IgM, ↑IgA, ↑IgE
PID 38	0.8y	M	CID	HALOPLEX PANEL 2		*NFKB1*	RECURRENT ESOPHAGEAL CANDIDIASIS, LUNG ABSCESS	ESOPHAGEAL ATRESIA	T+, B+, NK+
PID 39	13 mo	F	CID	HALOPLEX PANEL 1	***RAG1***	**	RECURRENT BRONCHITIS	SEVERE AUTOIMMUNE HEMOLITIC ANEMIA, INTERSTITIAL PNEUMOPATHY AND BRONCHIECTASIS	T+, B+, NK+
PID 40	na (adopted 11y)	F	CID	HALOPLEX PANEL 1			RECURRENT HERPETIC INFECTIONS (STOMATITIS)		↓T, ↓B, NK+
PID 41	18 mo	F	CID	HALOPLEX PANEL 1			RECURRENT RESPIRATORY INFECTIONS AND OTITIS, POSITIVE HCV	HODGKIN LYMPHOMA, OBSTRUCTIVE LUNG DISEASE	↓T (↓CD4), B-, ↑IgM, ↓IgG, ↓IgA
PID 42	1d	F	SYNDROMIC T-CELL DEFECT	ION TORRENT PANEL 1-2/ HALOPLEX PANEL 1			POST SURGICAL SEPSIS	CHDs, OSTEOMYELITIS	T-, B+, NK+
PID 43	8y	M	SYNDROMIC T-CELL DEFECT	ION TORRENT PANEL 1-2			URI, NEONATLA SEPSIS	ATOPY, CHDs	T+, B-, NK+
PID 44	4y	M	SYNDROMIC T-CELL DEFECT	ION TORRENT 1-2/ HALOPLEX PANEL 1			URI; SINUSITIS	MALFORMATIVE SYNDROME; PSYCHOMOTOR RETARDATION	T+, ↓B, NK+
PID 45	13y	M	UNCLASSIFIED T-CELL DEFICIENCY	ION TORRENT PANEL 2/HALOPLEX PANEL 2			CHRONIC EBV VIREMIA, PNEUMONIA	HEPATOSPLENOMEGALY, LYMPHOADENOPATY; NEPHROTIC SYNDROME	T (↓NAIVE CD4, CD8, RTE), ↓B, NK+, ↑IgM, ↓IgA
PID 46	3y	M	UNCLASSIFIED T-CELL DEFICIENCY	ION TORRENT PANEL 1-2/HALOPLEX PANEL 1			CHRONIC EBV VIREMIA, URI, LRI	PULMONARY NLH	T (↓NAIVE CD4, AND CD8), ↑B, ↑NK
PID 47	8y	M	UNCLASSIFIED T-CELL DEFICIENCY	ION TORRENT PANEL 1-2			CHRONIC EBV VIREMIA, URI, UTI	GASTROENTERITIS, ATOPIC DERMATITIS	T+ (↑CD4 CM) B+ NK+
PID 48	5y	M	UNCLASSIFIED T-CELL DEFICIENCY	ION TORRENT PANEL 1-2				AIHA; VASCULITIS, APHTOSIS	T (↓CD4) ↓B+ ↑NK
PID 49	6y	M	HIGM	ION TORRENT PANEL 1-2	***CD40LG***	**	CRYPTOSPORIDIUM	CHRONIC GASTRITIS,SCLEROSIS CHOLANGITIS	T+, B+(↓ SWITCHED MEMORY), NK+
PID 50	2y	M	HIGM	ION TORRENT PANEL 1		*CD40LG*	CHRONIC EBV VIREMIA	NEPHROTIC SYNDROME,PSYCHOMOTOR DELAY, LEUKODYSTROPHY	T+ (↑CD8 EM), B+(↓ SWITCHED MEMORY), NK+
PID 51	10y	M	AGAMMAGLOBULINEMIA	ION TORRENT PANEL 1 HALOPLEX PANEL 1	***RAG1***	**	CHRONIC EBV VIREMIA, URI	NASAL POLYPOSIS, CHRONIC BRONCOPNEUMOPATHY	T+, VERY ↓B, NK+, ↓IgM, ↓IgG, ↓IgA
PID 52	14y	M	CVID	ION TORRENT PANEL 1-2-3/ HALOPLEX PANEL 2			URI, PNEUMONIA	CHRONIC BRONCOPNEUNOPATY, BRONCHIECTASIS, GROWTH RETARDATION	T+(↓ NAIVE CD4 and CD8), ↓B (↓ SWITCHED MEMORY) , NK+, ↓IgM, ↓IgG, ↓IgA
PID 53	1y	F	CVID	ION TORRENT PANEL 1-2-3			UTI, PNEUMONIA	ATOPY	T+ (↑CD8 EM EMRA), ↓B (↓ SWITCHED MEMORY) , NK+, ↓IgG, ↓IgA
PID 54	5y	F	CVID	ION TORRENT PANEL 1-2-3		*PLCG2*	URI, PARASITE INFECTION (OXYURIASIS)		T+ B+ NK+, ↓IgM, ↓IgA
PID 55	7y	M	CVID	ION TORRENT PANEL 1-2-3			URI	GASTROENTERITIS	T+ (↑CD8), B+, NK+, ↓IgM, ↓IgG, ↓IgA
PID 56	14y	F	CVID	ION TORRENT PANEL 1-2-3		*CTLA4 + PTEN*	URI		T+ (↑CM, ↑THF, ↓TREG) B+ (↑NAIVE, ↓SWITCHED MEMORY, ↑AUTOREACTIVE B cells) NK+, ↓IgA
PID 57	1y	M	CVID	HALOPLEX PANEL 2		TNFRSF13B^*^+ *TCF3*	URI	NON-SPECIFIC COLITIS, NF1	T+ (↑CD4 CM), B+, NK+, ↓IgM, ↓IgG, ↓IgA
PID 58	1y	M	CVID	ION TORRENT PANEL 1/HALOPLEX PANEL 2		TNFRSF13B^*^+ *TCF3*	URI	NON-SPECIFIC COLITIS, NF1, ARTHITIS	T+ (↑CD4 CM), B+, NK+, ↓IgM, ↓IgG, ↓IgA
PID 59	5y	M	CVID	ION TORRENT PANEL 1-2-3			CHRONIC EBV VIREMIA, PNEUMONIA		T+ (↑γδ) B+,NK+, ↓IgA
PID 60	12y	F	CVID	HALOPLEX PANEL 2			CHRONIC EBV VIREMIA, URI, PNEUMONIA, WARTS		T+, ↓B, NK+, ↓IgA
PID 61	9mo	M	CVID	HALOPLEX PANEL 2		TNFRSF13B^*^	URI, LRI, HHV6	GASTROENTERITIS,ESSENTIAL ARTERIAL HYPERTENSION, ARNOLD-CHIARI SYNDROME TYPE I, GLICOSURIA, PSYCHOMOTOR DELAY	T+, B+(↓ SWITCHED MEMORY) NK+, ↓IgA
PID 62	2y	M	CVID	HALOPLEX PANEL 2				CHRONIC DIARRHEA, GASTROENTERITIS	T+ B+ (↑IgM MEMORY), NK+, HYPOGAMMAGLOBULINEMIA
PID 63	2y	M	CVID	HALOPLEX PANEL 2			UTI, LTI	MILD NEURODEVELOPMENTAL DELAY; DYSGENESIS OF THE CORPUS CALLOSUM; ARACHNOID CYST, MILD THROMBOCYTOPENIA	T+, LOW B, NK+ ↓IgM, ↓IgA
PID 64	11y	M	CVID	ION TORRENT PANEL 1-2-3					T+ (↑CD4 CM), B+(LOW SWITCHED MEMORY), NK+, ↓IgM, ↓IgG, ↓IgA
PID 65	2y	F	CVID	ION TORRENT PANEL 1-2-3			UTI, LTI (PNEUMOCOCCUS),	ECZEMATOUS DERMATITIS; GENERALIZED LYMPHADENOPATHY; HEPATOSPLENOMEGALY, GLILD	T+ (↓NAIVE CD4, ↑ EM CD8), B+(ABSENT MEMORY), NK+, ↓IgM, ↓IgG, ↓IgA
PID 66	3mo	F	CVID	ION TORRENT PANEL 1-2-3			URI; LRI; SEPSI	CANDIDA ENTERITIS,MALFORMATIVE SYNDROME, PSYCHOMOTOR RETARDATION, CHDs;CGH ARRAY: 15q25.1 DUPLICATION	T+ (↑EMRA CD4), B+, NK+, ↓IgM, ↓IgA
PID 67	15y	M	CVID	HALOPLEX PANEL 1			SALMONELLA OSTEOMIELYTIS	LINFOADENOPATHY, SPLENOMEGALY, AHA„ITP	T+, B+, NK+, HYPOGAMMAGLOBULINEMIA
PID 68	6y	M	CVID	HALOPLEX PANEL 2				LINFOADENOPATHY, SPLENOMEGALY, AHA, ITP, PULMONARY INFILTRATES, BRONCHIECTASIS	T+, B+, NK+, IgA-, IgG-
PID 69	1y	M	CVID	HALOPLEX PANEL 2			RECURRENT VZV, RECURRENT INFECTIONS	URTICARIA, ANGIOEDEMA, LUNG FIBROSIS	T+, B+, NK+, IgA ↓, IgG ↓
PID 70	15y	M	CVID	HALOPLEX PANEL 2		*TNFRSF13B*	RECURRENT INFECTIONS	LINFOADENOPATHY, SPLENOMEGALY,HYPOTHYROIDISM, LUNG NODULAR INFILTRATES, GROUND GLASS	T+, B+, NK+, IgM ↓
PID 71	12y	F	CVID	HALOPLEX PANEL 2			RECURRENT PNEUMONIA		T+, B+, NK+, HIPER IgG
PID 72	13y	M	CVID	HALOPLEX PANEL 1				PULMONARY NODULES	T+, B+, NK+, IgG-, IgM-, IgA-
PID 73	10y	M	SELECTIVE IgM DEFICIENCY	ION TORRENT PANEL 1-2-3			SEPSI; URI, LRI	GASTROENTERITIS; HEPATOSPLENOMEGALY	T+ (↑γδ), B+ ↓ MEMORY), NK+, ↓IgM
PID 74	12y	F	HYPERIGG4	HALOPLEX PANEL 2					T+, B+, NK+, ↑IgG4
PID 75	2y	M	UNCLASSIFIED ANTIBODY IMMUNODEFICIENCY	ION TORRENT PANEL 1-2-3		*TCF3*	ATYPICAL MYCOBACTERIOSIS (M.AVIUM)	BRONCHIAL GRANULOMA	T+ (↑CD4), B+, ↓NK+, ↓IgA
PID 76	5y	M	UNCLASSIFIED ANTIBODY IMMUNODEFICIENCY	ION TORRENT PANEL 1-3/HALOPLEX PANEL 2			CHRONIC HHV-6 VIREMIA, URI; PNEUMONIA; MOLLUSCUM CONTAGIOSUM	DERMATITIS	T+ (↑NAIVE CD4, ↑LATE EFFETOR CD8, ↓THF, ↓TREG) B+ (↓MEMORY, ↑TRANSITIONAL), NK+
PID 77	6y	F	UNCLASSIFIED ANTIBODY IMMUNODEFICIENCY	ION TORRENT PANEL 1-2-3/ HALOPLEX PANEL 2		*NOD2*	CHRONIC CMV AND HHV-6 VIREMIA	BURKITT LYMPHOMA, EBV-REACTIVATION, CMV PRIMARY INFECTION	T+ (LOW NAIVE CD8), B+ (LOW MEMORY), ↑IgM, ↓IgG, ↓IgA
PID 78	12y	M	UNCLASSIFIED ANTIBODY IMMUNODEFICIENCY	ION TORRENT PANEL 1-2-3/HALOPLEX PANEL 2			CHRONIC EBV VIREMIA	THROMBOCYTOPENIA; GASTROENTERITIS	T+, B+ (↓ IgM MEMORY AND ↓SWITCHED MEMORY), NK+, ↓IgM, ↓IgA
PID 79	3y	M	UNCLASSIFIED SYNDROMIC DEFICIENCY	ION TORRENT PANEL 1-2-3				THROMBOCYTOPENIA IMMUNOMEDIATED, CELIAC DISEASE	T+, B+, NK+, ↓IgA
PID 80	6y	F	IMMUNE DYSREGULATION	ION TORRENT PANEL 1			URI, LRI, RECURRENT SKIN INFECTIONS	DERMATITIS, FEMORAL DYSPLASIA	T+ (↑NAIVE CD4), B+ (↓ MEMORY) , NK+
PID 81	10y	M	IMMUNE DYSREGULATION	ION TORRENT PANEL 1-3/HALOPLEX PANEL 2		AIRE^*^+ PLCG2	URI	ALOPECIA; ONYCHODYSTROPHY	T+, ↓B+, NK+, ↓IgG, ↓IgA
PID 82	2y	F	INNATE IMMUNE DISEASE	ION TORRENT PANEL 1-2/HALOPLEX PANEL 1	***MYD88/CARD9***	**	CHRONIC EBV, HHV-6, CMV VIRMEMIA, URI; UTI; PBI	INGUINAL ABSCESS; GRANULOMATOUS LYMPHADENITIS	T+ B+ NK+
PID 83	3y	M	NEUTROPENIA	HALOPLEX PANEL 2	***JAGN1***	**	CHRONIC EBV VIREMIA, LRI,URI	APHTOSIS	T+, B+ (↓ IgM MEMORY and ↓SWITCHED MEMORY), NK+, ↑IgA
PID 84	1y	F	NEUTROPENIA	HALOPLEX PANEL 2	***CECR1***	**	RECURRENT INFECTIONS	SEVERE NEUTROPENIA	T+, B+, NK+, NEUTROPENIA
PID 85	5y	F	NEUTROPENIA	HALOPLEX PANEL 2				CARDIOPATHY, NEUTROPENIA, NEUROLOGICAL DELAY, LIGAMENT LAXITY	T+, B+, NK+, NEUTROPENIA
PID 86	13d	M	ALPS-LIKE	HALOPLEX PANEL 1	***NRAS***	**	URI	THROMBOCYTOPENIA; SPLENOMEGALY	T+ (↑CD4 CM, ↓RTE, ↑CD8 EM and ↑EMRA), B+(↓SWITCHED MEMORY), NK+
PID 87	3y	M	ALPS	HALOPLEX PANEL 2		*TNFRSF13B*	GENITAL AND PERIANAL WARTS, TONSILLITIS, PNEUMONIA	AHA, ITP, LINFOADENOPATHY, SPLENOMEGALY, HYPOGAMMAGLOBULINEMIA, PULMONARY INFILTRATES	T+, B+, NK+, ↓IgG, IgM-, ↓IgA
PID 88	8y	M	VEO-IBD	ION TORRENT PANEL 1	***XIAP***	**	CHRONIC EBV AND HVV-6 VIREMIA, URI	ENTEROPATHY	T+, B- (↑CD8 EM and ↑EMRA), NK+
PID 89	4y	M	VEO-IBD	ION TORRENT PANEL 1			CHRONIC EBV VIREMIA	ENTEROPATHY; CELIAC SPRUE	↓T+, ↑B+, NK+
PID 90	2y	M	VEO-IBD	ION TORRENT PANEL 1-2/HALOPLEX PANEL 2			CHRONIC VZV VIREMIA, URI	CHRONIC DIARRHEA, CELIAC SPRUE	T+ (↓NAIVE CD4) B+ NK+
PID 91	2mo	M	AUTOINFLAMMATORY SYNDROME	ION TORRENT PANEL 1-2	**	**	HLH; HEPATOSPLENOMEGALY; SKIN RASH; SYSTEMIC INFLAMMATORY SYNDROME	CHRONIC DIARRHEA, MONOCYTOPENIA	T+ (↑CM CD4+ ↓ RTE), B+ (↑SWITCHED MEMORY B CELL, ↑PLASMABLAST ↑CD21LOW, ↓TRANSITIONAL B CELL), AND DC-
PID 92	13y	F	AUTOINFLAMMATORY SYNDROME	HALOPLEX PANEL 2				SLE	T+, B+, NK+
PID 93	5y	F	UNCLASSIFIED SYNDROMIC DEFICIENCY	ION TORRENT PANEL 1-2			CHRONIC EBV VIREMIA, URI, PNEUMONIA	CHRONIC BRONCOPNEUNOPATY, MALFORMATIVE SYNDROME PSYCHOMOTOR DELAY	T+ (↑CM CD4+ ↑THF), B+ (↓ IgM MEMORY and ↓SWITCHED MEMORY), ↓ NK
PID 94	1y	M	UNCLASSIFIED SYNDROMIC DEFICIENCY	ION TORRENT PANEL 1-2			CHRONIC EBV VIREMIA	LAMBERT EATON SYNDROME, GLIOMA, 5q- MYELODISPLASIA; PSYCHOMOTOR RETARDATION; POLYNEUROPATHY	T+ ↓B NK+
PID 95	1.5y	M	UNCLASSIFIED SYNDROMIC DEFICIENCY	HALOPLEX PANEL 1		*BMP4*	URI, LRI	THROMBOCYTOPENIA, HYPERLAXITY, DENTAL ANOMALIES; DYSMORPHIC FEATURES; CRYPTORCHIDISM; SEVERE MYOPIA; ECTODERMAL DYSPALSIA SIGNS	T+, B+, NK+
PID 96	9y	M	SYNDROMIC	ION TORRENT PANEL 1-2			URI, POLYALLERGY	INTERSTITIAL TUBULOPATHY; CHRONIC PANCREATITIS; CHRONIC GASTRODUODENITIS; MILD ESOPHAGITIS; BRONCOPNEUMOPATHY WITH BRONCHIECTASIAS.	T+ (↑CM CD4+ ↓ RTE), B+, NK+
PID 97	4y	M	SYNDROMIC	ION TORRENT PANEL 1				HYPOSURRENALISM; COATS DISEASE; MYELODYSPLASIA; HYPOSPADIAS; MONOSOMY CHR 7	T+ (↑ CD4+), ↓B (↓TRANSITIONAL and ↑PLASMACELLS, NK+,↑IgA
PID 98	2y	M	ACUTE LIVER FAILURE	ION TORRENT PANEL 1-2			CHRONIC EBV VIREMIA, TWO EPISODES OF ACUTE EPATITIS	GROWTH RETARDATION, IUGR	T(↑ CD4+), B+, ↓NK+
PID 99	4y	M	HYPERSENSITIVITY	ION TORRENT PANEL 1			LTI (RECURRENT BRONCHITIS), ORAL PAPILLOMATOSIS, ATOPIC DERMATITIS	FOOD ALLERGY	T+ (↑γδ), B+, NK+
PID 100	16y	F	IMMUNE DYSREGULATION	HALOPLEX PANEL 1			RECURRENT INFECTIONS	ENTEROCOLITIS	T+, B+, NK+
PID 101	7y	F	IMMUNE DYSREGULATION	HALOPLEX PANEL 2			WARTS, NAIL FUNGAL INFECTION (NOT RECURRENT)	ALOPECIA, AUTOIMMUNE THYROIDITIS, MILD LYMPHOPENIA	↓T, B+, NK+
PID 102	na	M	OTHER (TROMBOCYTOPENIC PURPURA)	ION TORRENT PANEL 1-2				HYPOSPADIAS, ITP	T+ (↑CM CD4+), B+, NK+
PID 103	11y	M	OTHER	HALOPLEX PANEL 2				ALOPECIA	T+, B+, NK+
PID 104	13y	M	OTHER	HALOPLEX PANEL 2				ITP	T+, B+, NK+, ↓IgG, ↓IgA
PID 105	4y	F	OTHER	HALOPLEX PANEL 2				AUTOIMMUNE/AUTOINFLAMMATORY PHENOTYPE	

*EBV, Epstein-Barr; CMV, Cytomegalovirus; VZV, Varicella-Zoster Virus; HHV-6, Human Herpesvirus 6; HPV, Human Papilloma Virus; URI, Upper Respiratory Infection; LRI, Lower Respiratory Infections; UTI, Urinary Tract Infection; SLE, Systemic Lupus Erythematosus; ITP, Idiopathic Thrombocytopenic Purpura; HLH, Hemophagocytic Lymphohistiocytosis; AIHA, Autoimmune Haemolytic Anemia; CHDs, Congenital Heart Disease ; NF1, Neurofibromatosis 1. ↓ low as compared to age matched normal range; ↑high as compared to age matched normal range*.

Black Bold: Ion Torrent diagnosis. Blue Bold: Haloplex diagnosis. Different colors show genes in which we found: Violet

* *> Previous Sanger detections in predisposing gene variants to PID; Violet > predisposing gene variants to PID. Gray > no-causative disease variants; Green > variants of uncertain significance (VUS). Orange > variant in genes partially associated to the clinical phenotype*.

### Ion Torrent Target System

#### Panel Design

The construction of targeted panels design required the study of several reported clinical phenotypes of known PID genes described in the IUIS (International Union of Immunological Societies) in the years 2014–2015. Our three custom Ion Torrent panels were designed with Ampliseq Designer software using GRCh37 (panel 1 and 2) and GRCh38 (panel 3) as references. Primers were divided into two pools. The first custom panel (panel 1) contains 17 known genes related to SCID-CID phenotypes (85.85 kb). The second custom panel (panel 2) includes 24 genes for less frequent CID phenotypes (101.9 kb) and the third panel (panel 3) includes 62 genes for CVID (240.01 kb) (Supplementary Tables S1–S3). The final design was expected to cover 95.43% of the first panel, 94.13% of the second panel and 97.2% of the third genes panel. For each gene included in the panels a 10 bp of exon padding was included to cover the flanking regions of exon's coding sequences (CDS) including (panel 1 and 2) or not (panel 3) the untranslated regions (UTRs).

#### Ion Torrent Gene Target Library Preparation and NGS Sequencing

DNA was extracted by QIAamp DNA Blood Mini Kit (Qiagen). Five nanograms of gDNA were used for library preparation. DNA was amplified with 17 amplification cycles using gene panel Primer Pools and AmpliSeq HiFi mix (Thermo Fisher). PCR pools for each sample were combined and subjected to primer digestion with FuPa reagent (Thermo Fisher). Libraries were indexed using the Ion Xpress Barcode Adapter Kit. After purification, the amplified libraries were quantified with Qubit® 2.0 Fluorometer. All samples were diluted at a final concentration of 100 pM, then amplicon libraries were pooled for emulsion PCR (ePCR) on an Ion OneTouch System 2TM using the Ion PGM Template OT2 200 kit or Ion Chef according to manufacturer's instructions. Quality control of all libraries was performed on Qubit® 2.0 Fluorometer. Ampliseq Design Samples were subjected to the standard ion PGM 200 Sequencing v2 protocol using Ion 316 v2 chips or Ion S5 using Ion 520 v2 chips (Life Technologies).

#### Ion Torrent Bioinformatics Analysis, Variants Filtering, and Assessment of Pathogenicity

Mapping and variants calling were performed using the Ion Torrent suite software v3.6. Sequencing reads were aligned on GRCh37 (panel 1 and 2) and GRCh38 (panel 3) reference genome using the program distributed within the Torrent mapping Alignment Program (TMAP) map4 algorithm (Thermo Fisher; https://github.com/Ion Torrent/TS). The alignment step is limited only to the regions of target genes. BAM files with aligned reads were processed for variant calling by Torrent Suite Variant Caller TVC program and variants in Variant Calling Format (VCF) file were annotated with ANNOVAR. Called variants with minimum coverage of 30X, standard Mapping Quality and Base Phred Quality were examined on Integrative Genome Viewer (IGV) and BIOMART. Filtering procedures selected variants with a minor allele frequency (MAF) < 2% annotated using the following public databases: 1000 Genomes Project (2500 samples; http://www.1000genomes.org/), the Exome Variant Server (ESP) (6500 WES samples; http://evs.gs.washington.edu/EVS/) and the Exome Aggregation Consortium (ExAC) (60,706 samples; http://exac.broadinstitute.org/). Nonsense, frame-shift, start lost, stop lost, and canonical splice site variants were considered potentially pathogenic (6). *In silico* prediction of functional consequences of novel SNV was performed using Mutation taster, LTR, Polyphen2, SIFT, and CADD score >15 (26–30) and literature available data. Supplementary Figure 1A summarizes all steps of the process.

### Haloplex Target System

#### Panel Design

We designed *two* panels including up to 300 known PID genes (3) chosen from a Custom Gene Target Panel from Agilent SureDesign online tool (http://web16.kazusa.or.jp/rapid_original/) and about 300 candidate additional genes taken from the RAPID web site (http://rapid.rcai.riken.jp) from the RIKEN Center for Integrative Medical Science, from the literature and the ESID Online Registry. The candidate genes category includes genes that might be found in clinically relevant PID pathways and can share similar biological function of known PID genes. The first panel of 623 target genes comprised 7,245 regions with 66,600 amplicons, while the second panel of 601 target genes, included 6,984 regions and 73,061 amplicons. The designed probes capture 25 flanking bases in the coding exons regions (Supplementary Tables 4A,B). The final probe design was expected to cover >97% of target regions. Practical coverage is indicated.

#### Haloplex Gene Target Library Preparation and NGS Sequencing

Genomic DNA was extracted by QIAamp DNA Blood Mini Kit (Qiagen) and quantified by Qubit dsDNA BR Assay Kit (Thermofisher). DNA integrity was check by agarose gel (1% of agarose in TAE 1x). Genomic DNA was enriched with Haloplex Target Enrichment System kit (Agilent Technologies Inc., 2013, Waghäusel-Wiesental, Germany). Libraries were prepared according to the manufacturer's instructions. Briefly, 225 ng of genomic DNA was enzymatically digested; fragments were hybridized with conjugated biotin probes for 16 h at 54°C. Circularized target DNA-Haloplex probe hybrids were captured with streptavidin-coated magnetic beads. DNA ligase was added to the capture reaction to close nicks in the circularized probe-target DNA hybrids. All DNA samples were individually indexed during the hybridization step and library PCR amplification was performed on the Mastercycler Nexus Thermal Cyclers (Life Sciences Biotechnology, Hamburg, Germany). Amplicons were purified with AMPure XP beads (Beckman Coulter, Inc., Krefeld, Germany). Sequencing was performed with a MiSeq Reagent Kit v3 (600 Cycles) with 7 pM of sample libraries loaded on the Illumina MiSeq (San Diego, CA, USA). Quality controls after fragmentation and final concentration of prepared libraries, were assessed by Bioanalyzer (Agilent Technologies Inc., Eindhoven, the Netherlands).

#### Haloplex Bioinformatics Analysis, Variants Filtering, and Assessment of Pathogenicity

FastQ files were aligned to the human reference genome (UCSC hg19, GRCh37) by Burrows–Wheeler Aligner (31). Picard HsMetrics was applied to analyze the target-capture sequencing experiments (http://picard.sourceforge.net/) and internal scripts were used to calculate mean gene coverage. Variant calling was performed by Freebayes (32). Raw variants were filtered by the following parameters: QUAL> 1, (QUAL/AO)> 10, SAF> 0, SAR > 0, RPR > 1, RPL > 1. Variants with an allele depth below 20 reads were excluded from the analysis. Selected variants were annotated for dbSNP-146, ClinVar, dbNSFP v2.9 databases and SnpEff (33) and were filtered for Common Allele Frequencies (CAF) < 5% and variant effect on exons (missense, frameshift, splice acceptor/donor, start lost, stop lost, stop gained, 3′UTR, 5′UTR). Variants found in the either 5′ or 3′ UTR were excluded from the subsequent analyses. *In silico* analysis for variants' pathogenicity was determined according to 5 prediction tools: Mutation taster, LTR, Polyphen2, SIFT, and CADD score >15 (26–30). In case of trios, variants were subdivided according to model of inheritance (Autosomal Recessive/Dominant, X-linked, *De novo*). The complete bioinformatics analysis is reported in Supplementary Figure 1B.

### Statistical Analysis

Data were analyzed with Graph-Pad Prism, version 6.2 (Graph Pad Software, la Jolla, CA).

## Results

### Characterization of PID Patients

**Figure 1:**
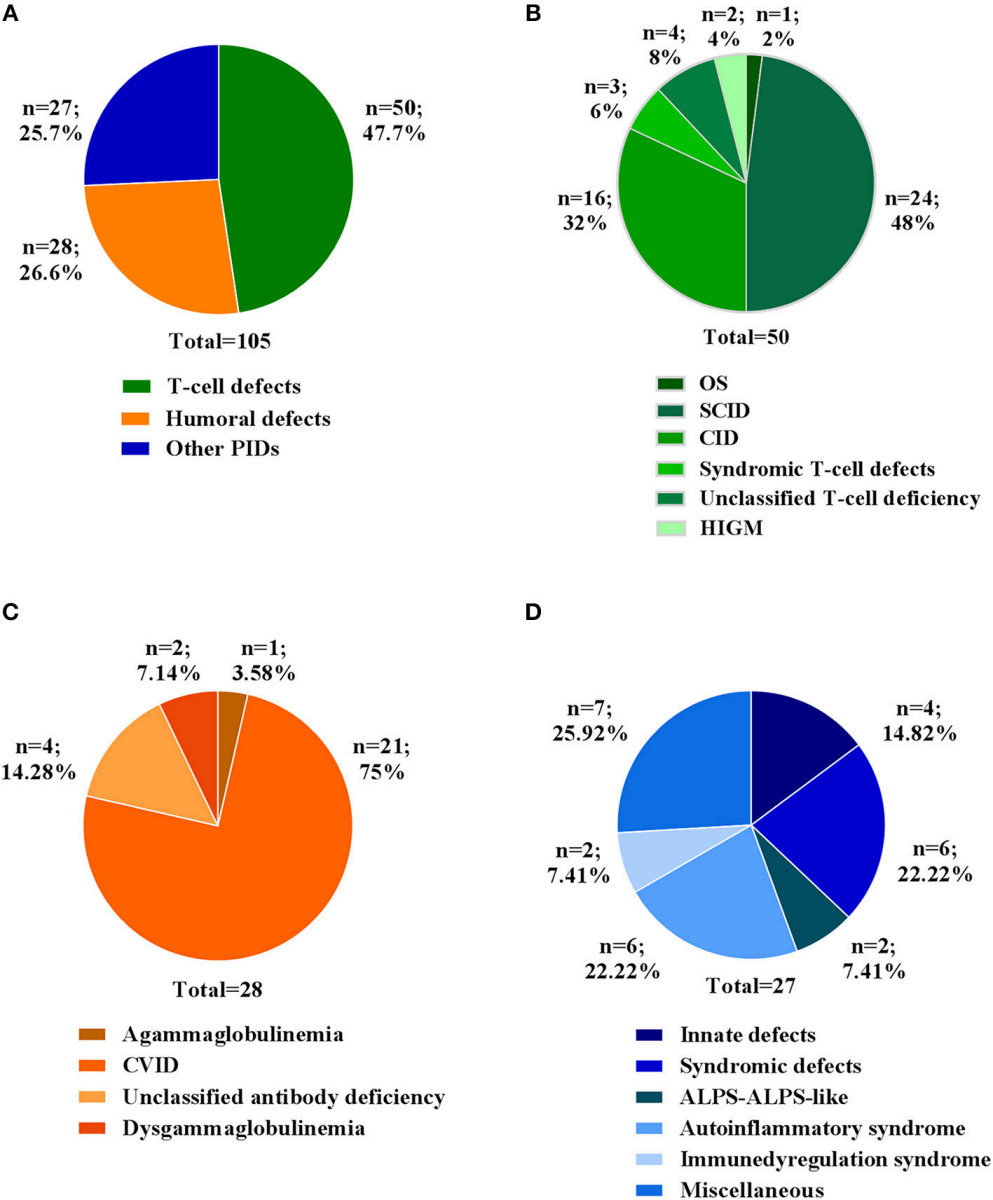
Clinical diagnosis of patients at admission. **(A)** Percentage of patients for three main categories. Percentage of each clinical diagnosis in patients belonging to **(B)** *T cell defects*, **(C)** *Humoral defects* and **(D)** *Other PIDs* categories. For each category the total number of patients is indicated.

In this study, we report the clinical and molecular characterization of 105 PID patients presenting with either typical or overlapping PID phenotypes. Patients were clustered according to initial clinical presentation in 3 main categories (Figure 1A): *T-cell defects* (including Omenn syndrome, SCID, CID, syndromic T-cell defect, unclassified T-cell deficiency, hyper IgM syndrome); *Humoral defects* (agammaglobulinemia, CVID, unclassified antibody deficiency, dysgammaglobulinemia); *Other PIDs* (immune dysregulation, innate immunity defects including congenital defects of phagocytes, syndromic defects with immune-deficiency signs/symptoms, ALPS-ALPS-like, autoinflammatory syndrome, and a miscellaneous that includes non-typical PID patients with a broad range of clinical phenotypes). The clinical, immunological, and molecular features are reported in Table 1. The percentage of patients in each subgroup is shown in Figures 1B–D. Among the *T-cell defects* (*n* = 50; 47,7%), the majority of patients presented with SCID (48%), followed by CID (32%) (Figure 1B). The *Humoral Defects* group (*n* = 28; 26,6%) was mainly represented by CVID (75%), while the *Other PIDs* group (*n* = 27; 25,7%) included a wide spectrum of rare defects and uncommon phenotypes.

**Figure 2:**
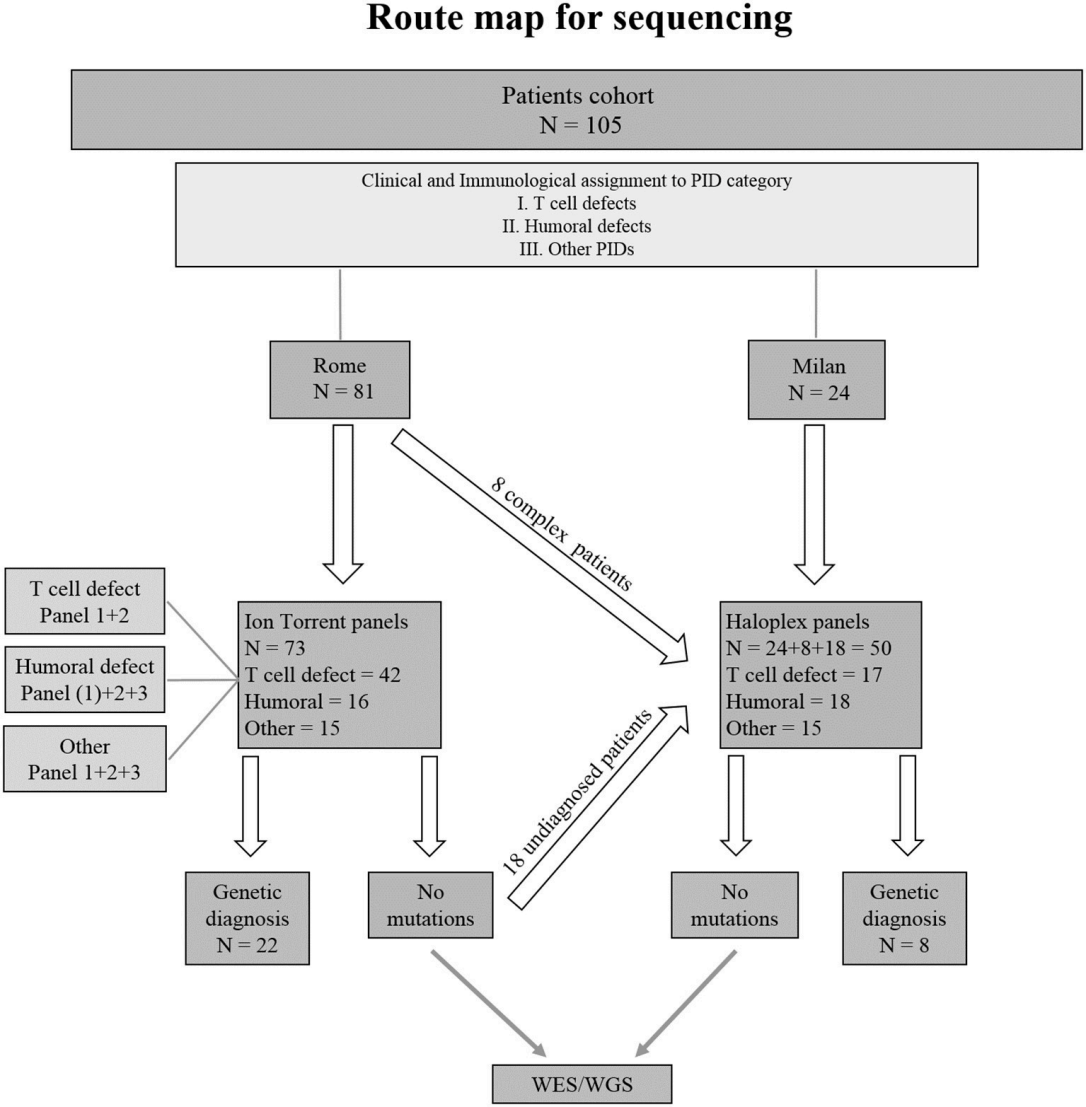
Flowchart indicating the strategy of the study. (1) Indicates the only patient in *Humoral defect* group who has been analyzed by Ion Torrent panel 1.

Seventy-three PID patients were analyzed by Ion Torrent sequencing system using three different panels including SCID/CID and CVID known genes. Two Haloplex panels including more than 600 known and candidate PID genes were applied to 32 additional patients. Additionally, 18 patients previously analyzed by Ion Torrent but still without a clear molecular diagnosis, were analyzed by Haloplex system. A flow chart showing the *route map* for sequencing of index patients is shown in Figure 2.

### Target Enrichment Performance and Gene Coverage

The mean target coverage resulted of 529 ± 169X (panel 1), 361 ± 97X (panel 2) and 417 ± 117X (panel 3) for Ion Torrent and 229 ± 25X for Haloplex panels (Supplementary Figure 2A). The mean target coverage for Ion Torrent panels was optimal as compared to recently published works in which a coverage of 335X was obtained (34). Indeed, the Ion Torrent expected coverage of the coding regions was 95.43% for panel 1 (SCID-CID), 94.13% for panel 2 (rare CID) and 97.2% for the panel 3 (Supplementary Tables 1–3). The practical coverage obtained from Ion Torrent panels is shown in Supplementary Figures 2B–D.

Primer design for Haloplex aimed at covering more than 97% of the coding regions for all genes. The observed coverage of the targeted regions after running the two panels is represented in Supplementary Tables 4A,B. The majority of shared genes included in all panels and analyzed by both technologies were well-covered (Supplementary Figures 3A–C).

### Performance Evaluation

The use of large panels for NGS retrieved a big number of data as compared to small panels. Putative variants detected by Ion Torrent have been examined and validated obtaining an average of false positive variants < 0.6%. Such value decreases reducing the number of genes included in the panel. Haloplex produces larger amount of variants, but only the ones significantly indicative among those related to the patient's phenotype have been investigated; hence, we could not properly evaluate data accuracy. In the 18 patients resequenced by Haloplex, no variants in genes included in the Ion Torrent panels were found supporting the accuracy of these methods. Furthermore, 6 available samples previously diagnosed by Sanger sequencing with 8 known different mutations in *RAG1, IL2RG, JAK3*, and *LIG4* genes, were included in the study and detected by Ion Torrent panel 1 (Table 2A).

**Table 2A:** Genetic mutations in 6 positive control PID patients.

**ID**	**Disease**	**Gene**	**RefSeq**	**Mutation**		**dbSNP and references**	**Zygosity**	**Method**	**OMIM**
PID I	OS	*RAG1*	NM_000448	a) c.1682G>A; p.R561H	b) c.1871G>A; p.R624H	rs104894284; rs199474680	Compound Heterozygous	Ion Torrent	OMIM ^*^179615
PID II	SCID	*IL2RG*	NM_000206	a) c.452T>C; p.L151P		rs137852511	Hemizygous	Ion Torrent	OMIM ^*^308380
PID III	SCID	*JAK3*	NM_000215	**a) c.1208G>A; p.R403H**		Scarselli et al. (35)	Homozygous	Ion Torrent	OMIM ^*^600173
PID IV	SCID	*LIG4*	NM_001352601	a) c.833G>A, p.R278H	b) c.1271_1275delAAAGA; p.K424RfsTer20	Cifaldi et al. (36)	Compound Heterozygous	Ion Torrent	OMIM ^*^601837
PID V	leaky SCID	*RAG1*	NM_000448	a) c.2521C>T; p.R841W		rs104894287	Homozygous	Ion Torrent	OMIM ^*^179615
PID VI	leaky SCID	*RAG1*	NM_000448	a) c.256_257del; p.K86VfsTer33		rs772962160	Homozygous	Ion Torrent	OMIM ^*^179615

*In bold novel mutations*.

One false negative diagnosis has been recently recognized. Indeed, the Torrent Suite Variant Caller TVC program was unable to identify the c.C664T: p.R222C mutation in exon 5 of IL2RG gene in patient PID16 but this was detectable on IGV.

### Molecular Diagnoses

**Figure 3:**
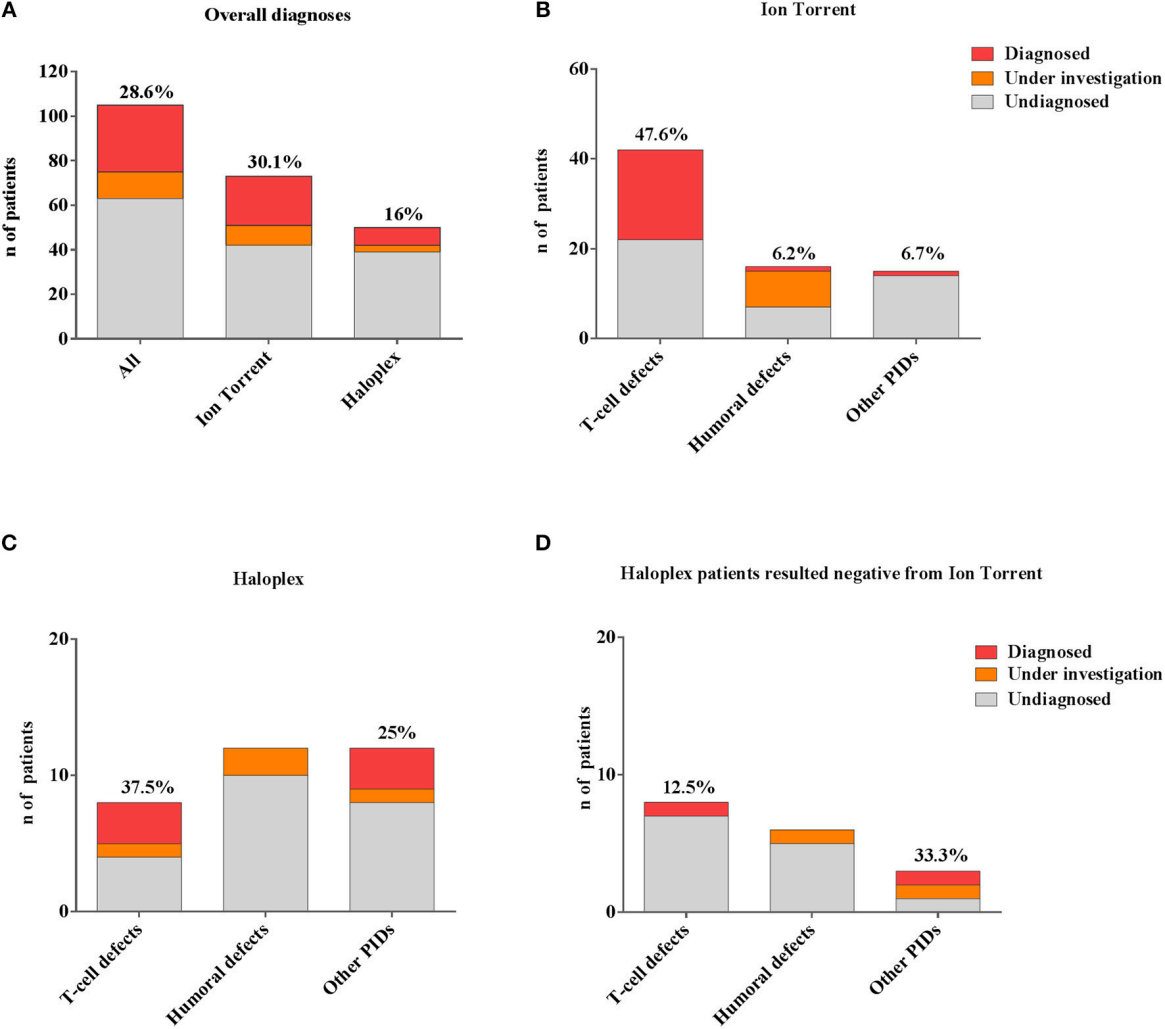
Comparison between different number of genetic diagnoses obtained by Ion Torrent and Haloplex. **(A)** Histogram showing the number of overall *diagnosed* (red), *under investigation* (orange) or *undiagnosed* (gray) patients. **(B)** Histogram showing the Ion Torrent diagnoses. **(C)** Histograms showing Haloplex diagnoses. **(D)** Diagnostic findings by Haloplex in negative Ion Torrent patients. The percentages refer only to diagnosed patients.

In our cohort, 28.6% (30/105) of molecular diagnosis was obtained (Figure 3A). Sanger sequencing for all mutations and parents' carrier status were performed. Functional studies were conducted for most novel variants and results are reported in Table 2B.

**Table 2B:** Mutations detected in our PID cohort.

**ID**	**Disease**	**Gene**	**RefSeq**	**Mutation**		**dbSNP and references**	**Zygosity**	**Inheritance**	**Method**	**OMIM**	**Functional test**
PID 1	OS	*RAG1*	NM_000448	a) c.1870C>T; p.R624C	b) c.2521C>T; p.R841W	rs199474688; rs104894287	Compound Heterozygous	Familial	Ion Torrent	OMIM ^*^179615	
PID 2	SCID	*RAG2*	NM_000536	a) c.685C>T; p.R229W		rs765298019	Homozygous	Unknown	Ion Torrent	OMIM ^*^179616	
PID 3	SCID	*RAG2*	NM_000536	**a) c.1A>G; p.M1V**	b) c.1403_1406del ATCT	n.d.; rs786205616	Compound Heterozygous	Familial	Ion Torrent	OMIM ^*^179616	n.a.
PID 4	SCID	*RAG1*	NM_000448	a) c.1681C>T; p.R561C	**b) c.1815G>C; p.M605I**	rs104894285; Dobbs et al. (37)	Compound Heterozygous	Familial	Ion Torrent	OMIM ^*^179615	Recombinase activity ongoing
PID 5	SCID	*IL2RG*	NM_000206	a) c.202G>A; p.E68K		rs.1057520644	Hemizygous	Familial	Ion Torrent	OMIM ^*^308380	
PID 6	SCID	*JAK3*	NM_000215	**a) c1796T>G; p.V599G**	b) c.2125T>A; p.W709R	Di Matteo et al. (38); rs748216175	Compound Heterozygous	Familial	Ion Torrent	OMIM ^*^600173	Published data
PID 12	SCID	*ADA*	NM_000022	**a) c. 455T>C p.L152P**	b) c.478+6T>C	n.d.; Santisteban et al. (39)	Compound Heterozygous	Familial	Ion Torrent/Haloplex	OMIM ^*^608958	Reduced ADA enzymatic activity
PID 15	SCID	*ADA*	NM_000022	**a) c.367delG; p.D123fsTer10**		n.d.	Homozygous	Familial	Ion Torrent	OMIM ^*^608958	Reduced ADA enzymatic activity
PID 16	CID	*IL2RG*	NM_000206	a) c.C664T:p.R222C		rs111033618	Hemizygous	De novo	Ion Torrent	OMIM ^*^308380	
PID 17	SCID	*IL2RG*	NM_000206	a) c.677G>A; p.R226H		rs869320660	Hemizygous	Familial	Ion Torrent	OMIM ^*^308380	
PID 18	SCID	*IL2RG*	NM_000206	a) c.854G>A; (splice)		rs111033617	Hemizygous	Familial	Ion Torrent	OMIM ^*^308380	
PID 19	SCID	*IL2RG*	NM_000206	**a) c.455T>G; p.V152G**		n.d.	Hemizygous	Familial	Ion Torrent	OMIM ^*^308380	n.a.
PID 20	SCID	*RAG1*	NM_000448	a) c.1229G>A; p.R410Q	**b) c.1863delG; p.A622QfsTer9**	rs199474684; n.d.	Compound Heterozygous	Unknown	Ion Torrent	OMIM ^*^609889	n.a.
PID 21	SCID	*CD3D*	NM_000732	a) c.274+5G>A		rs730880296	Homozygous	Mother; n.a.	Ion Torrent	OMIM ^*^186790	
PID 22	SCID	*RAG1*	NM_000448	**a) c.987delC: p.S330LfsTer15**		n.d.	Homozygous	Unknown	Ion Torrent	OMIM ^*^179615	Evident pathogenicity
PID 23	SCID	*JAK3*	NM_000215	a) c.308G>A; p.R103H		rs774202259	Homozygous	Unknown	Ion Torrent	OMIM ^*^600173	
PID 24	SCID	*JAK3*	NM_000215	a) c.1132G>C; p.G378R	b) c.1442-2A>G	rs1485406844; JAK3base_D0095	Compound Heterozygous	Familial	Ion Torrent	OMIM ^*^600173	
PID 25	SCID	*IL7R*	NM_002185	**a) c.134A>C; p.Q45P**	b) c.537+1G>A	n.d.; rs777878144	Compound Heterozygous	Familial	Haloplex	OMIM ^*^146661	n.a.
PID 29	CID	*RAG1*	NM_000448	a) c. 2521C>T; p.R841W		rs104894287	Homozygous	Familial	Ion Torrent	OMIM ^*^179615	
PID 31	CID	*RAG1*	NM_000448	a) c.1871G>A; p.R624H	**b) c. 1213A>G; p.R405G**	rs199474680; n.d.	Compound Heterozygous	Familial	Ion Torrent	OMIM ^*^179615	Recombinase activity ongoing
PID 36	CID	*IL7R*	NM_002185	**a) c.160T>C; p.S54P**	**b) c.245G>T; p.C82F**	rs1002396899; rs757797163	Compound Heterozygous	Familial	Ion Torrent	OMIM ^*^146661	
PID 37	CID	*ARPC1B*	NM_005720	**a) c.64+1G>A**		Brigida et al. (40) (accepted)	Homozygous	Familial	Haloplex	OMIM ^*^604223	Published data
PID 39	CID	*RAG1*	NM_000448	**a) c.2119G>C; p.E665D**	b) c.519delT ; p.Glu174SerfsTer27	n.d.; rs1241698978	Compound Heterozygous	Unknown	Haloplex	OMIM ^*^179615	n.a.
PID 49	HIGM	*CD40LG*	NM_000074	a) c.410-2 A>T		rs1254732497	Hemizygous	Familial	Ion Torrent	OMIM ^*^300386	
PID 51	CVID	*RAG1*	NM_000448	a) c.1871G>A; p.R624H	**b) c.2182T>C; p.Y728H**	rs199474680; Cifaldi et al. (41)	Compound Heterozygous	Familial	Ion Torrent	OMIM ^*^179615	Published data
PID 88	IBD	*XIAP*	NM_001167	**a) c.566T>C; p.L189P**		Cifaldi et al. (42)	Hemizygous	De novo	Ion Torrent	OMIM ^*^300079	Published data
PID 83	NEUTROPENIA	*JAGN1*	NM_032492	a) c.63G>T; p.E21D		rs587777729	Homozygous	Familial	Haloplex	OMIM ^*^616012	
PID 84	NEUTROPENIA	*CECR1*	NM_001282225	**a)c.1367A>G, p.Y456C**	b)c.1196G>A, p.W399^*^	Barzaghi et al. (43)	Compound Heterozygous	Familial	Haloplex	OMIM ^*^607575	Accepted for publication
PID 82	INNATE IMMUNE DISEASE	*CARD9*	NM_052813	a) c.1434+1G>C	Chiriaco et al. (44)	rs141992399	Homozygous	Familial	Ion Torrent/ Haloplex	OMIM ^*^607212	
		*MYD88*	NM_002468	a) c.195_197delGGA; p.E66del		rs878852993	Homozygous	Familial		OMIM ^*^602170	
PID 86	ALPS-LIKE	*NRAS*	NM_002524	a) c.35G>A; p.G12D		rs121913237	Heterozygous	Somatic	Haloplex	OMIM ^*^164790	

In bold novel not described mutations

A rapid molecular diagnosis was established in 30.1% (22/73) of PID patients who were investigated by Ion Torrent. Diagnoses were achieved in *RAG1, RAG2, IL2RG, JAK3, ADA, CD3D, IL7R, CD40L*, and *XIAP* genes (see Table 2B). As expected, the identification of a molecular defect resulted more frequent in patients with a clear clinical and immunological phenotype as shown in those included in the group of *T cell defects* (20/42; 47.6%) (Figure 3B). Interestingly, the percentage of diagnosis in the group of SCID/CID patients was 60.6% (20/33).

The percentage of molecular diagnosis for the 50 patients studied through the Haloplex panels was of 16% (8/50) as shown in Figure 3A. The first 6 diagnoses were obtained in a cohort of 32 patients. Three SCID/CID patients with mutations in RAG1, IL7R and ARPC1B genes [(40, 45–47) and Volpi et al., under revision] were diagnosed in 8 *T cell defects* (37,5%). Moreover, *JAGN1* (48)*, CECR1* (43) and *NRAS* genes, associated to complex phenotypes, were identified in 12 of the *Other PIDs* group (25%) (Figure 3C).

Two additional patients were diagnosed analyzing the 18 patients, previously negative by Ion Torrent, presenting with a less defined immunological phenotype (Figure 3D). For one patient (PID12), the Ion Torrent panel 1 was able to detect only a missense mutation in the *ADA* gene. Haloplex identified the second intronic mutation located in the fifth nucleotide upstream exon 5, not included in the Ion Torrent design, of the gene. In the second Ion Torrent negative patient (PID82) presenting an atypical HyperIgE syndrome, Haloplex detected two rare homozygous mutations in *MYD88* and *CARD9* genes, which were not included in the Ion Torrent panels (1 and 2). The pathogenic role of each single gene mutation is still under investigation but this molecular information is important to optimize the clinical management of the patient including the evaluation of HSCT as definitive treatment (44).

In summary, 4 SCID/CID patients out of a total of 16 *T cell defects*, were identified by Haloplex, demonstrating once more a higher percentage of diagnosis in this PID group (Table 2B). However, although the possibility to identify a causative gene mutation correlates with a precise clinical clusterization, the identification of patients, with complex and extended phenotypes, needs larger NGS panels.

### Disease-Associated Variants

**Figure 4:**
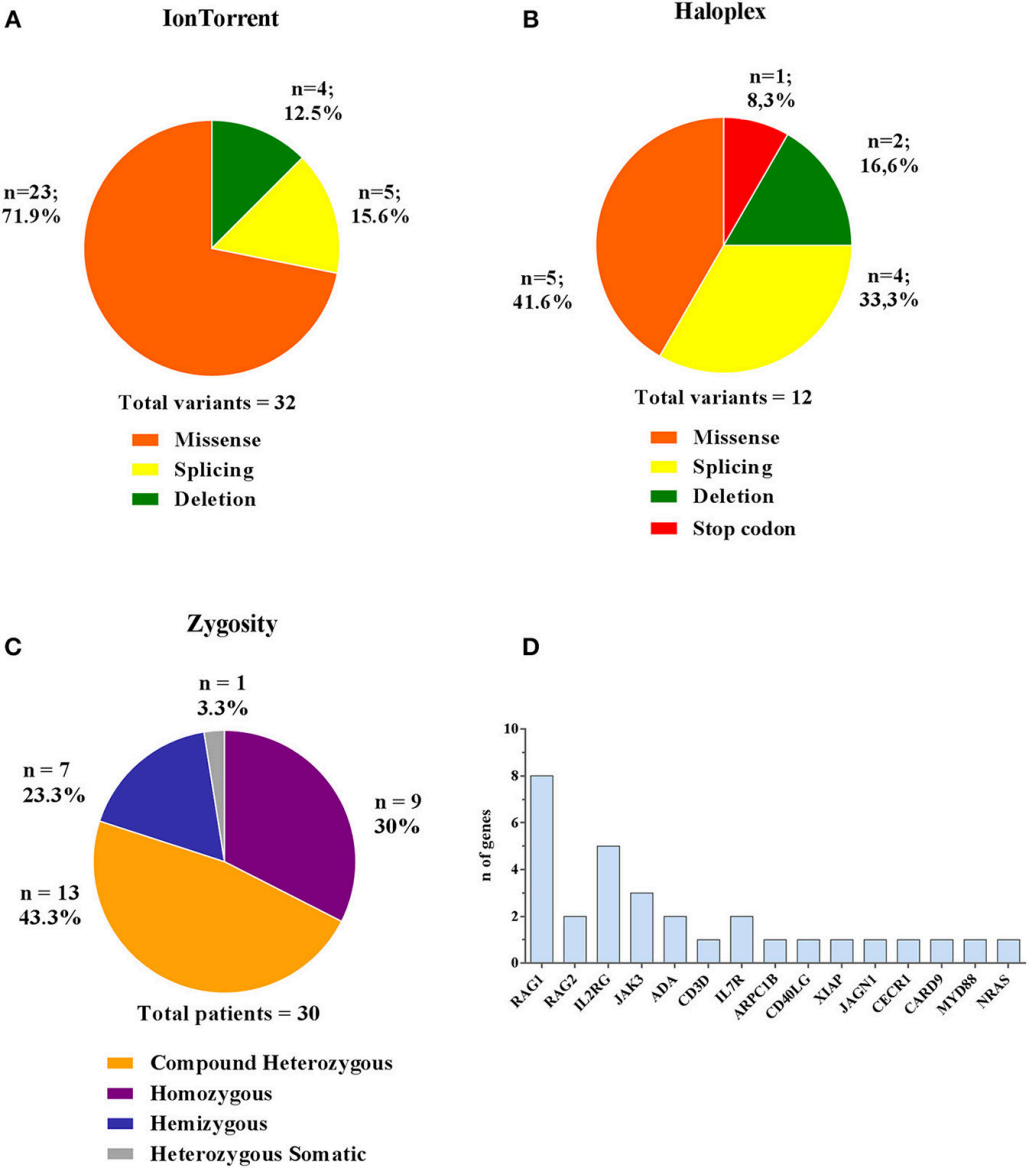
Type and zygosity of mutations and mutated genes distribution. Types and percentage of mutations found in diagnosed PID patients for Ion Torrent **(A)** and Haloplex **(B)**. **(C)** Overall observed zygosity for diagnosed PID patients. **(D)** Total number of detected mutated genes.

Comparing the results obtained by the two methods, 44 (32 Ion Torrent and 12 Haloplex) disease-associated variants have been identified in 30 patients, of whom 18 were novel (Table 2B). The majority of variants detected by Ion Torrent were missense (*n* = 23; 74.2%) as summarized in Figure 4A. We were also able to detect 4 small deletions and 5 splice site variants. The Haloplex panels detected 5 missense, 2 deletions, 4 splice site and 1 stop codon variants (Figure 4B). Among the 30 diagnosed patients, we found 13 compound heterozygous patients with mutations in *RAG1, JAK3, ADA, IL7R*, and *CECR1* genes, 9 homozygous variants including *ADA, RAG1, RAG2, CD3D, JAK3, ARPC1B, MYD88/CARD9*, and *JAGN1*, 7 hemizygous variants in *IL2RG, CD40LG*, and *XIAP*, and only 1 heterozygous somatic variant in *NRAS* (Figure 4C). Therefore, most patients enrolled in this study were offspring of non-consanguineous marriages. The most frequent mutated gene in our cohort is *RAG1* followed by *IL2RG* (Figure 4D).

### Putative Neutral Variants vs. Variants of Uncertain Significance (VUS)

Fifteen CVID patients were initially analyzed by Ion Torrent panels 1-2, but no causative variants were found. We therefore designed a specific CVID panel and found 4 putative causative variants suggestive of AD disease that was confirmed by Sanger sequencing. Indeed, we found a heterozygous damaging variant in the *CTLA4* gene and a predicted damaging variant in the *PTEN* gene in an adult patient followed since childhood (PID56). The patient inherited one mutation from the father and one from the mother but the real role of these variants and their possible combined effect is still under investigation. In addition, two other VUS in *TCF3* and *PLCG2* genes were found in two patients (PID75 and PID54), in which no other evidences are available (see Table 1).

A rare variant in *CD40L* gene (p.R200S) found in patient PID50 was excluded from the analysis, although an altered CD40L expression was detected. This variant was predicted benign in multiple databases. Furthermore, a homozygous rare variant in *CECR1* gene (p.Q233R) was found in patient PID13. However, the two proband's healthy brothers were found to be homozygous for this variant thus it was not considered pathogenic, nevertheless, additional functional studies will be performed to exclude genetic predisposition (e.g., ADA2 activity, protein expression).

Three novel variants of uncertain significance (VUS) identified by Haloplex in patients with classical and complex phenotypes are still “under investigation.” We are currently validating a novel damaging variant in the *TCF3* gene in two twin patients (PID57-58) and their mother affected by CVID (49). EMSA assay is ongoing to assess the capacity of TCF3 protein to bind DNA target sequences. In these twin patients we also previously found by Sanger sequencing a mutation in TNFRSF13B gene already described to be associated to CVID (50).

A causative variant in the *BMP4* gene (51) with a severe myopia, ectodermal dysplasia, and cytopenia was found in a patient (PID95) in whom the altered immunological phenotype remains poorly explained by this mutation. Moreover, *NF*κ*B1* variant in a CID patient (PID38) was found but its significance is still under investigation.

Finally, heterozygous variants in *TNFRSF13B* (PID70, PID87) and *NOD2* (PID77), genes were found by Haloplex in three patients. Generally, variants in susceptibility genes involved in the disease pathogenesis should be considered for potential future phenotypic implications particularly in adult patients where multiple factors may contribute to the onset of the disease.

## Discussion

The application of multigene NGS panels has extended our knowledge of PIDs and is currently recognized as a comprehensive diagnostic method in the field of rare disorders consenting the diagnosis in the 15–70% of all cases depending on the PID clinical and phenotypic clusterization (25, 52). In the present work we show that the complementary, integrated use of two custom-made targeted sequencing approaches, Ion Torrent or Haloplex, allowed to clearly identify causative variants in 28.6% (*n* = 30) of the patients in all groups of PIDs, confirming the value of NGS assays to obtain a genetic diagnosis for PIDs (17–23).

The Ion Torrent approach resulted highly successful for SCID patients, a group generally more defined for its immunological and clinical presentation (53). Indeed, with this approach we identified 20/33 SCID/CID patients (60,6%). The Haloplex workflow was able to identify causative variants in 8/50 patients (16%) of whom 4 were found in the group of SCID/CID patients and 4 fall in that of complex and extended phenotypes. Interestingly, a molecular diagnosis was achieved in 2/18 (11%) patients presenting with typical and atypical clinical phenotypes resulted negative after Ion Torrent analysis and included in the Haloplex approach.

By NGS it is possible to identify unexpected mutations in apparently not corresponding PID cases, as recently reported by our group for a patient with agammaglobulinemia due to *RAG1* deficiency (41). This result strengthen the notion of a large phenotypic variety associated with RAG deficiency, suggesting that it should be considered also in patients presenting with an isolated marked B-cell defect (54–57) and as already reported that RAG mutations are more frequent than expected. Notably, RAG1 is the most frequent PID cause in our cohort. This case represents a paradigmatic model of how new questions arise on the management and follow-up for patients in which a milder phenotype could be associated to alternative treatments to transplantation (41, 57, 58).

CVID is a typical example of a disease with a broad phenotype due to different gene alterations (59–61). Notably, in 4 CVID patients with mutations in *TNFRSF13B* and *AIRE* previously detected by Sanger sequencing (see Table 1) we extended NGS analysis to looking for novel disease causing genes. Therefore, frequent variants comparable to polymorphisms should be considered with caution since the pathogenic meaning is still unclear. Additional functional studies in these cases are required. Four additional diagnoses are summarized in Supplementary Table 5 (62). These were obtained after the completion of the present study by other targeted NGS panels and Sanger sequencing, indicating that the combination of in-depth clinical knowledge and appropriate sequencing techniques can lead to new diagnoses.

Although the prioritization methods applied in this study follows all common assumptions for a correct data analysis, the identification of novel variants currently under investigation represents a challenge and their validation needs the essential support of further in-depth experimental studies (6–8, 63). The integration of clinical, immunological, biochemical and molecular data might favor a revised PIDs classification of patients with similar phenotype due to a different genetic cause, or patients with different phenotypes but with the same genetic cause. In our experience, the use of selected NGS panels is useful and easy to handle for rapid diagnosis in clinically and immunologically well-characterized phenotypes. As compared to WES, targeted small NGS panels provide an important alternative for clinicians for direct sequencing of relevant genes, guaranteeing a high coverage and sequencing depth (64). On the contrary, their application in patients with atypical phenotypes could result in an incomplete and delayed diagnosis. Extended gene panels or WES should be directly used in these cases for research purposes, to allow the diagnosis of unexpected genotype-phenotype association.

As reported by several groups (7, 8, 22–24), the application of targeted WES for each suspicion of PID by exploring gene-by-gene also for limited numbers of striking genes still remain time and resource consuming in the absence of synergy between clinical and bioinformatics supports. This is yet unfeasible for extended diagnostic purposes. Indeed, the huge amount of retrieved data and the risk of incidental findings in other non-PID genes involved in different monogenic or multifactorial pathologies may be confounding and do not corresponding to the first suspicion. Additionally, the confidence of the results decreases with the number of targeted genes and may preclude any variant detection in self-evident known genes (65). Many previously undetected variants do not have a well-defined role in our genome (1.5 × 10^6^ million variants in each genome and lesser in exome). In this scenario, ethical and legal issues related to the disclosure of genetic information generated by NGS need to be considered and guidelines should be developed to help the different specialists to translate the genetic results into the clinics (64).

The achievement of NGS application will require further integration of knowledge based on clinical, immunological and molecular data and the collaboration among different experts in these fields. A better clinical, immunological and genetic characterization of new PIDs will significantly contribute to the identification of diagnostic and prognostic markers and early individual therapeutic strategies with significant patients' benefit.

## Data Availability

Data have been uploaded to ClinVar, accession number: SUB5252744.

## Author Contributions

CrC, IB, and GD performed experiments, developed gene panels for targeted sequencing. CrC, IB, FB, and DMG interpreted the results and wrote the manuscript. DP, CrC, VF, FS, CaC, and GD created gene clusters to filter variants and integrated clinical and bioinformatics analysis of data retrieved by Ion Torrent platform. IB, DL, DC, FB, MPC, MZ, DP, CrC, GD, MO, and CaC created gene clusters to filter variants and integrated clinical and bioinformatics analysis of data retrieved by Haloplex workflow. CrC, IB, SD, GF, MC, MZ, MG, AV, and GD performed molecular and functional experiments. FB, MPC, EA, FC, AS, FL, FF, CP, GF, GB, PM, DM, ClC, PP, SC, AT, VM, LC, CA, AF, FLi, PR, CaC, and AA provided or referred clinical samples and patient's clinical data. GD, IB, SG, FS, CrC, CaC, and AA participate to the study design and data interpretation. CaC, FS, GD, and AA designed the research, participate to the study design and data interpretation. FS, VM, SG, SF, and FLi made substantial contributions to revising the manuscript. CaC, GD, and AA supervised the research and manuscript revision. Legend: CrC, Cifaldi Cristina; FC, Conti Francesca; CaC, Cancrini Caterina; ClC, Canessa Clementina; FL, Licciardi Francesco; FLi, Locatelli Franco.

### Conflict of Interest Statement

The authors declare that the research was conducted in the absence of any commercial or financial relationships that could be construed as a potential conflict of interest.

## Supplementary Material

Supplementary Figure 1 Schematic representation of filtering variants strategy for Ion Torrent **(A)** and Haloplex **(B)**.

Supplementary Figure 2 Coverage analysis. **(A)** Mean target coverage for genes included in Haloplex and Ion Torrent panels 1-2 and 3. Box and whiskers show median, 5th and 95th percentiles. Haloplex shows 605 shared genes in the two panels. **(B)** Mean gene coverage for Ion Torrent Panel 1, **(C)** panel 2 and **(D)** panel 3. Coverage is shown as number of reads.

Supplementary Figure 3 Comparison of coverage analysis. **(A–C)** Comparison of mean gene coverage in shared genes between Ion Torrent and Haloplex panels.

Supplementary Table 1 Theoretical coverage of genes included in Ion Torrent panel 1.

Supplementary Table 2 Theoretical coverage of genes included in Ion Torrent panel 2.

Supplementary Table 3 Theoretical coverage of genes included in Ion Torrent panel 3.

Supplementary Table 4 **(A)** Theoretical and effective coverage of gene intervals in Haloplex platform panel 1. **(B)** Theoretical and effective coverage of gene intervals in Haloplex platform panel 2.

Supplementary Table 5 Additional diagnoses obtained after this study.

## Abbreviations

AD: Autosomal Dominant
AR: Autosomal Recessive
CAF: Common Allele Frequency
CDS: Coding Sequence
CID: Combined Immunodeficiencies
CVID: Common Variable Immunodeficiency
IGV: Integrative Genome Viewer
MAF: Minor Allele Frequency
NGS: Next Generation Sequencing
PCR: Polymerase Chain Reaction
PID: Primary Immunodeficiency
SCID: Severe Combined Immunodeficiency
SNP: Single Nucleotide Polymorphism
UTR: Untranslated Region
VCF: Variant Calling Format
WES: Whole Exome Sequencing
WGS: Whole Genome Sequencing.
