# Corrigendum: Targeted NGS Platforms for Genetic Screening and Gene Discovery in Primary Immunodeficiencies

**DOI:** 10.3389/fimmu.2019.01184

**Published:** 2019-05-31

**Authors:** Cristina Cifaldi, Immacolata Brigida, Federica Barzaghi, Matteo Zoccolillo, Valentina Ferradini, Davide Petricone, Maria Pia Cicalese, Dejan Lazarevic, Davide Cittaro, Maryam Omrani, Enrico Attardi, Francesca Conti, Alessia Scarselli, Maria Chiriaco, Silvia Di Cesare, Francesco Licciardi, Montin Davide, Francesca Ferrua, Clementina Canessa, Claudio Pignata, Silvia Giliani, Simona Ferrari, Georgia Fousteri, Graziano Barera, Pietro Merli, Paolo Palma, Simone Cesaro, Marco Gattorno, Antonio Trizzino, Viviana Moschese, Loredana Chini, Anna Villa, Chiara Azzari, Andrea Finocchi, Franco Locatelli, Paolo Rossi, Federica Sangiuolo, Alessandro Aiuti, Caterina Cancrini, Gigliola Di Matteo

**Affiliations:** ^1^Unit of Immune and Infectious Diseases, University Department of Pediatrics (DPUO), Scientific Institute for Research and Healthcare (IRCCS) Childrens' Hospital Bambino Gesù, Rome, Italy; ^2^San Raffaele Telethon Institute for Gene Therapy (SR-Tiget), IRCCS San Raffaele Scientific Institute, Milan, Italy; ^3^Pediatric Immunohematology and Bone Marrow Transplantation Unit, Scientific Institute for Research and Healthcare (IRCCS) San Raffaele Scientific Institute, Milan, Italy; ^4^Department of Systems Medicine, University of Rome Tor Vergata, Rome, Italy; ^5^Department of Biomedicine and Prevention, University of Rome Tor Vergata, Rome, Italy; ^6^Vita Salute San Raffaele University, Milan, Italy; ^7^Center for Translational Genomics and BioInformatics, San Raffaele Scientific Institute, Milan, Italy; ^8^Division of Immunology and Rheumatology, Department of Paediatric Infectious Diseases, Regina Margherita Children's Hospital, University of Turin, Turin, Italy; ^9^Pediatric Immunology, Department of Health Sciences, University of Florence, Florence, Italy; ^10^Meyer Children's Hospital, Florence, Italy; ^11^Department of Translational Medical Sciences, University of Naples Federico II, Naples, Italy; ^12^Department of Molecular and Translational Medicine, A. Nocivelli Institute for Molecular Medicine, University of Brescia, Brescia, Italy; ^13^Unit of Medical Genetics, St. Orsola-Malpighi University Hospital, University of Bologna, Bologna, Italy; ^14^Division of Immunology Transplantation and Infectious Diseases (DITID), Diabetes Research Institute (DRI) IRCCS San Raffaele Scientific Institute, Milan, Italy; ^15^Pediatric Department, San Raffaele Scientific Institute, Milan, Italy; ^16^Department of Onco-Hematology and Cell and Gene Therapy, Scientific Institute for Research and Healthcare (IRCCS) Childrens' Hospital Bambino Gesù, Rome, Italy; ^17^Paediatric Hematology-Oncology, “Ospedale della Donna e del Bambino”, Verona, Italy; ^18^Center for Autoinflammatory Diseases and Immunodeficiencies, IRCCS Giannina Gaslini, Genoa, Italy; ^19^Department of Pediatric Hematology and Oncology, “ARNAS Civico Di Cristina Benfratelli” Hospital, Palermo, Italy; ^20^Pediatric Immunopathology and Allergology Unit, University of Rome Tor Vergata Policlinico Tor Vergata, Rome, Italy; ^21^Milan Unit, National Research Council (CNR) Institute for Genetic and Biomedical Research (IRGB), Milan, Italy; ^22^Humanitas Clinical and Research Institute, Rozzano, Italy; ^23^Department of Pediatric Hematology and Oncology, Scientific Institute for Research and Healthcare (IRCCS) Childrens' Hospital Bambino Gesù, University of Rome La Sapienza, Rome, Italy

**Keywords:** primary immunodeficiencies, Next Generation Sequencing, gene panels, Ion Torrent, Haloplex

In the published article, there was an error in affiliation “18.” Instead of “Pediatric and Rheumatology Unit, Giannina Gaslini Institute, Genoa, Italy,” it should be “Center for Autoinflammatory Diseases and Immunodeficiencies, IRCCS Giannina Gaslini, Genoa, Italy.”

The authors apologize for this error and state that this does not change the scientific conclusions of the article in any way. The original article has been updated.

