# Supplementary figures and images for: Targeted NGS Platforms for Genetic Screening and Gene Discovery in Primary Immunodeficiencies
Source: Front Immunol. 2019 Apr 11;10:316. doi: 10.3389/fimmu.2019.00316 (PMC6470723; doi:10.3389/fimmu.2019.00316)

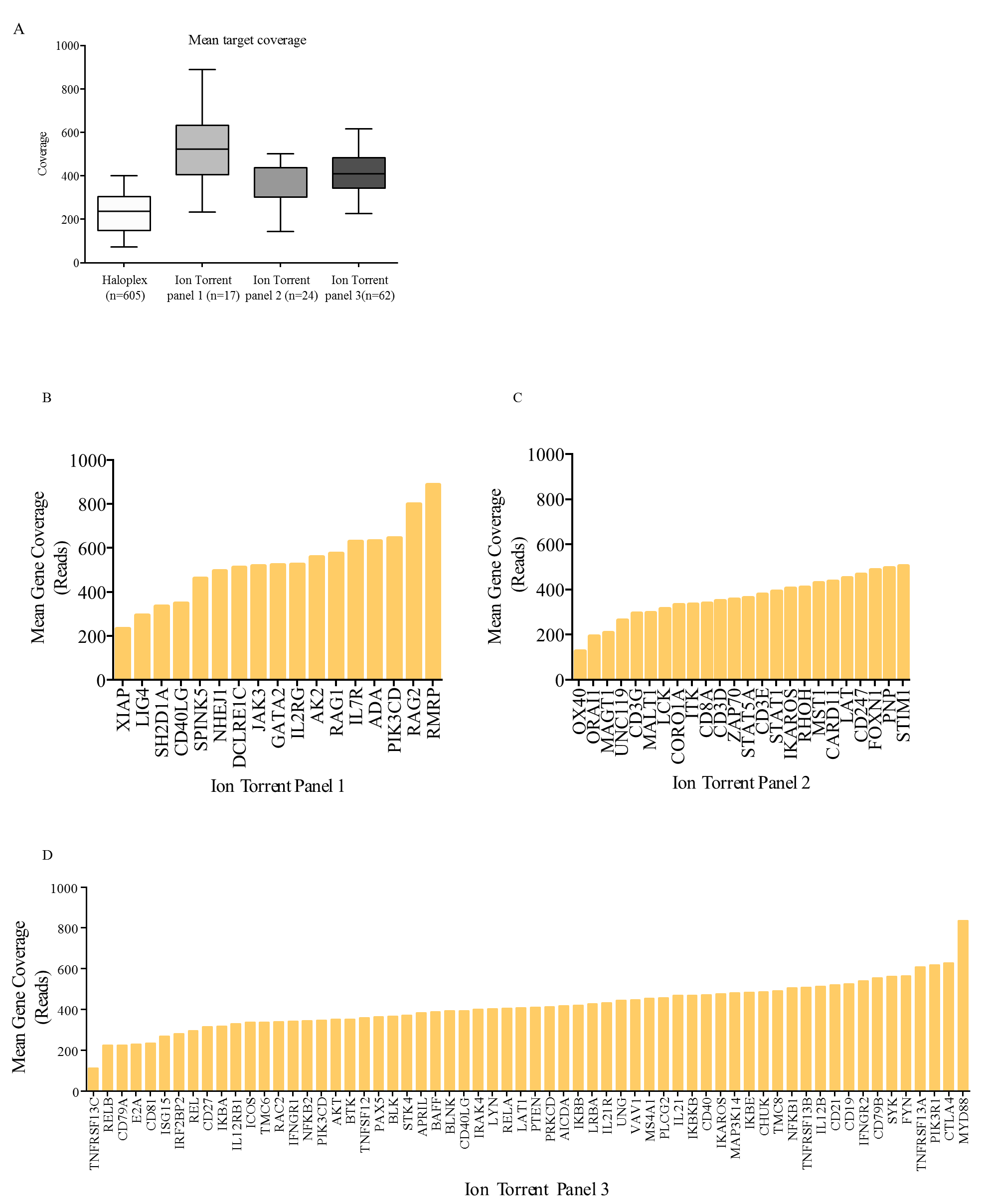

Supplement: Supplementary Figure 2 — Coverage analysis. (A) Mean target coverage for genes included in Haloplex and Ion Torrent panels 1-2 and 3. Box and whiskers show median, 5th and 95th percentiles. Haloplex shows 605 shared genes in the two panels. (B) Mean gene coverage for Ion Torrent Panel 1, (C) panel 2 and (D) panel 3. Coverage is shown as number of reads. [file Image_2.TIFF]

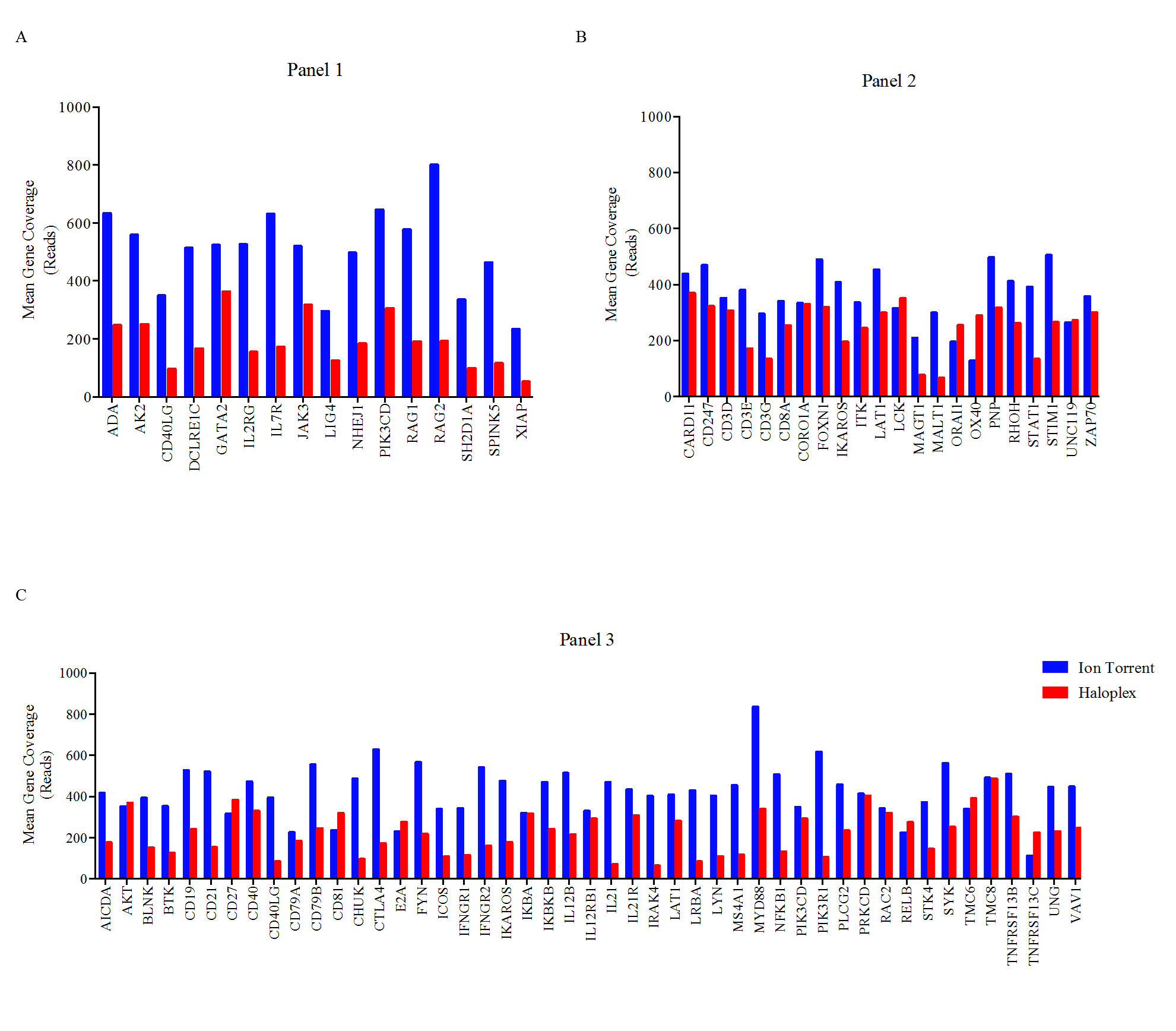

Supplement: Supplementary Figure 3 — Comparison of coverage analysis. (A–C) Comparison of mean gene coverage in shared genes between Ion Torrent and Haloplex panels. [file Image_3.TIFF]
